# The Impact of Artificial Intelligence in the Endoscopic Assessment of Premalignant and Malignant Esophageal Lesions: Present and Future

**DOI:** 10.3390/medicina56070364

**Published:** 2020-07-21

**Authors:** Daniela Cornelia Lazăr, Mihaela Flavia Avram, Alexandra Corina Faur, Adrian Goldiş, Ioan Romoşan, Sorina Tăban, Mărioara Cornianu

**Affiliations:** 1 Department V of Internal Medicine I, Discipline of Internal Medicine IV, “Victor Babeș” University of Medicine and Pharmacy Timișoara, Romania, Eftimie Murgu Sq. no. 2, 300041 Timișoara, Romania; lazar.daniela@umft.ro (D.C.L.); ioan.romosan@gmail.com (I.R.); 2 Department of Surgery X, 1st Surgery Discipline, “Victor Babeș” University of Medicine and Pharmacy Timișoara, Romania, Eftimie Murgu Sq. no. 2, 300041 Timișoara, Romania; 3 Department I, Discipline of Anatomy and Embriology, “Victor Babeș” University of Medicine and Pharmacy Timișoara, Romania, Eftimie Murgu Sq. no. 2, 300041 Timișoara, Romania; alexandra_pantu@yahoo.com; 4 Department VII of Internal Medicine II, Discipline of Gastroenterology and Hepatology, “Victor Babeș” University of Medicine and Pharmacy Timișoara, Romania, Eftimie Murgu Sq. no. 2, 300041 Timișoara, Romania; goldisadi@yahoo.com; 5 Department II of Microscopic Morphology, Discipline of Pathology, “Victor Babeș” University of Medicine and Pharmacy Timișoara, Romania, Eftimie Murgu Sq. no. 2, 300041 Timișoara, Romania; sorinataban@yahoo.com (S.T.); marioaracornianu@yahoo.com (M.C.)

**Keywords:** artificial intelligence, computer-assisted diagnosis, endoscopy, Barrett’s esophagus, esophageal cancer

## Abstract

In the gastroenterology field, the impact of artificial intelligence was investigated for the purposes of diagnostics, risk stratification of patients, improvement in quality of endoscopic procedures and early detection of neoplastic diseases, implementation of the best treatment strategy, and optimization of patient prognosis. Computer-assisted diagnostic systems to evaluate upper endoscopy images have recently emerged as a supporting tool in endoscopy due to the risks of misdiagnosis related to standard endoscopy and different expertise levels of endoscopists, time-consuming procedures, lack of availability of advanced procedures, increasing workloads, and development of endoscopic mass screening programs. Recent research has tended toward computerized, automatic, and real-time detection of lesions, which are approaches that offer utility in daily practice. Despite promising results, certain studies might overexaggerate the diagnostic accuracy of artificial systems, and several limitations remain to be overcome in the future. Therefore, additional multicenter randomized trials and the development of existent database platforms are needed to certify clinical implementation. This paper presents an overview of the literature and the current knowledge of the usefulness of different types of machine learning systems in the assessment of premalignant and malignant esophageal lesions via conventional and advanced endoscopic procedures. This study makes a presentation of the artificial intelligence terminology and refers also to the most prominent recent research on computer-assisted diagnosis of neoplasia on Barrett’s esophagus and early esophageal squamous cell carcinoma, and prediction of invasion depth in esophageal neoplasms. Furthermore, this review highlights the main directions of future doctor–computer collaborations in which machines are expected to improve the quality of medical action and routine clinical workflow, thus reducing the burden on physicians.

## 1. Introduction

Over time, machine learning (ML), a component of artificial intelligence (AI), has been implemented in a variety of medical specialties, such as radiology, pathology, gastroenterology, neurology, obstetrics and gynecology, ophthalmology, and orthopedics, with the goal of improving the quality of healthcare and medical diagnosis [1]. 

In clinical gastroenterology practice, due to technological developments, estimates show that AI could have the ability to create a predictive model; for instance, it could develop an ML model that can stratify the risk in patients with upper gastrointestinal bleeding [2,3], establish the existence of a specific gastrointestinal disease, define the best treatment, and offer prognosis and prediction of the therapeutic response [4,5,6]. In this context, by applying ML or deep learning (DL) (AI using neural networks), clinical management in gastroenterology can begin to focus on more personalized treatment centered on the patient and based on making the best individual decisions, instead of relying mostly on guidelines developed for a specific condition. Moreover, the goal of implementing these AI-based algorithms is to increase the possibility of diagnosing a gastrointestinal disease at early stage or the ability to predict the development of a particular condition in advance [7]. 

Because both AI and gastroenterology encompass many subdomains, the interaction between them might take on various forms. In recent years, we have witnessed a large explosion of research in attempts to improve various fields of gastroenterology, such as endoscopy, hepatology, inflammatory bowel diseases, and many others, with the aid of ML. We also note that, because of the requirement to diagnose more patients with gastrointestinal cancers at an early stage of the disease, which is associated with curative treatment and better prognosis, many studies were developed to address improvement of the detection of these tumors with the aid of AI. 

Numerous studies have been performed, using AI to improve the detection of early neoplasia developed on the background of Barrett’s esophagus [8,9] and early esophageal squamous cell carcinoma [10].

This paper offers an overview of the most prominent research data on endoscopic assessment of premalignant and malignant esophageal lesions with the aid of AI. Our review highlights the advantages and drawbacks of these new algorithms based on ML in the field of gastroenterology and supplies insight into new perspectives on collaboration between physicians and computers and future applications of this technology in gastroenterology practice.

## 2. Methods

A literature search modality was applied for all English language literature published in the last 15 years, before June 2020, by assessing the PubMed electronic database. The keywords used for our research purposes were “esophageal cancer”, “esophageal neoplasm”, “Barrett esophagus”, “artificial intelligence”, “machine learning”, “deep learning”, “convolutional neural network”, “detection”, and “diagnosis”. The specific search was also performed to identify clinical studies involving AI for the endoscopic evaluation of Barrett esophagus/esophageal cancer, using the ClinicalTrials.gov and University Hospital International Network Clinical trial Registry (UMIN-CTR) database. 

## 3. Definitions of Artificial Intelligence Terminology

### 3.1. Artificial Intelligence (AI)

The concept of AI was first mentioned in the 1950s [11] and refers to the capacity of a computer to perform tasks that might mimic the human mind, including “cognitive” functions such as the abilities of “learning” and “problem solving” [12].

### 3.2. Machine Learning (ML)

The term ML, introduced for the first time in 1959 by Arthur Samuel from the IBM company, refers to an IT domain whereby a computer system can acquire the ability to “learn” by using data without specific programming and can therefore develop a predictive mathematical algorithm based on input data, using recognition of “features”. The ML “model” is subsequently able to adapt to new situations in which it becomes able to predict and make decisions.

**Figure 1:**
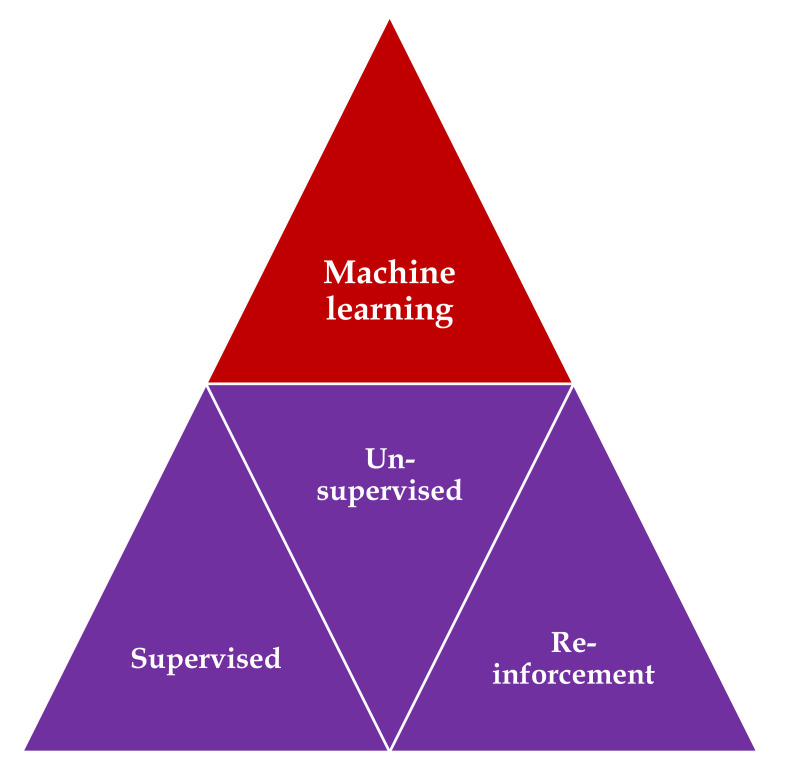
Types of machine learning algorithms: supervised learning—task driven (classification); unsupervised learning—data driven (clustering); and reinforcement learning—algorithm learns from trial and error.

Three main types of learning methodologies are recognized, namely, *supervised learning*, in which the computer learns from familiar patterns; *unsupervised learning*, in which the computer discovers the common aspects in unknown patterns; and, finally, *reinforcement learning*, in which the computer has the ability to learn from trial and error [13,14] (Figure 1). *Clustering algorithms* are based on unsupervised learning, in which unlabeled data self organizes to predict outcomes (e.g., clustering). *Classification and regression algorithms* are based on unsupervised learning, in which prelabeled data train a model to predict new outcomes. *Rewards and recommendations algorithms* are based on reinforcement learning, which gives feedback to an algorithm when it does something right or wrong. 

The predictive models encompass the key elements of the “training”, “validation”, and “testing” datasets. Approximately 70% of samples are commonly used in the initial training set to develop the model, and the remaining 30% of the samples are used as model validation and testing sets, but these percentages may vary with the application [7]. 

AI was implemented in the medical field, using different types of ML, such as binary classifiers, Bayesian inferences, decision trees, ensemble trees, linear discriminants, support vector machines (SVM), k-nearest neighbors, logistic regression, and artificial neural networks (ANNs) [15,16]. 

Support vector machine (SVM), which was invented in 1963, before the development of DL [17], represents a supervised learning model, a discriminative algorithm that uses a dividing hyperplane. SVM demonstrated its best accuracy in classification and regression analysis.

#### 3.2.1. ML Using Hand-Crafted Features (Conventional Algorithms)

For a long time, ML using images (in the field of gastroenterology, we are using endoscopic images) relied primarily on hand-crafted features. In this context, IT specialists coded a mathematical description of specific patterns, such as color and texture. The researchers manually indicated the potential features of the images based on clinical expertise. A classifier was trained to distinguish between different classes of features, and eventually, the model was able to use this knowledge to recognize the class in a new set of images [14].

#### 3.2.2. ML Using Deep Learning (DL)

DL refers to a subset of ML techniques that is built from multiple-layered neural network algorithms. They represent ML algorithms that use layers of nonlinear processing for “feature extraction”, which is the selection of powerful predictive variables, and “transformation”, which refers to changing the data for more efficient construction of the model [18].

##### Neural Networks 

Neural networks represent a specific area of ML that shows similarities with the human brain, namely densely interconnected neurons, with the aim of recognizing specific patterns, extracting features, or learning different characteristics of the training dataset to elaborate a concrete result [7,18]. 

Therefore, in the case of an artificial neural network (ANN), we use a fully connected neural network in which the outputs of the neurons of one layer represent the input for the neurons of the next layer. Each connection has a specific weight that is learned during the training process, and the model is based on a nonlinear sigmoidal function.

A deep neural network represents an ANN containing several hidden layers between the input and output layer. This technology proved to possess excellent accuracy for establishing diagnosis and predicting prognosis in the medical area. In most cases, DL outperforms the hand-crafted algorithm, but it requires a larger quantity of data for learning [19,20]. Fortunately, most of the initial weaknesses and limits of the deep neural network have been overcome by the recent availability of big data for training and the major progress in computing software power [15,16]. 

One drawback of DL is its “black-box” nature, meaning that the system cannot apply reason to the machine-generated decision, which can be a confusing aspect for the endoscopist. However, a new research area known as “interpretable DL” has attracted recent attention through its attempt to present an argument-based framework for decision-making [21]. Although DL models proved to be the most performant algorithms in fitting the data, one of their limits is their dependency on the training dataset. This “overfitting” error appears if the training database is not sufficiently diverse or contains bias. In that case, the results might not be validated and implemented in real-life circumstances. To enlarge the training datasets, these approaches might include images showing normal aspects and images containing pathologic lesions. Additionally, most of the recent studies use augmentation of the image-based data by resizing and cropping of the frame, with a subsequent flipping along the axis [4,16]. 

Convolutional neural networks (CNN) represent a specific class of ANN composed of convolutional and pooling layers with the role of extracting specific features and fully connected layers that fulfill the task of elaborating the definitive classification. 

**Figure 2:**
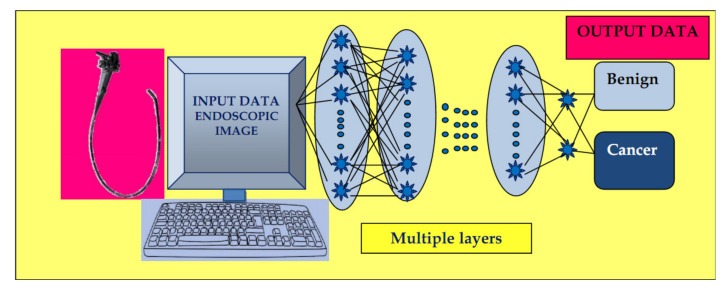
Convolutional neural network (CNN) system: input layer with raw data of the endoscopic image, multiple layers with the role of extracting specific features, and elaborating image classification in the output layer.

The input images are subjected to the preprocessing procedure of filtering (convolution) to extract specific features. Subsequently, the convolution filter undergoes a learning process to elaborate the performant feature maps, which are compressed to smaller sizes. At the end, the fully connected layers combine the selected features for design of the final model. In case of CNN, the number of weights is significantly lower than that of the fully connected networks. The CNN has demonstrated excellent performance in image analysis (Figure 2). 

The concept of CNN was developed independently by several different groups during the 1970s and 1980s. The proof of concept of CNN emerged in the late 1980s when Bengio, Hinton, and LeCun started to exchange ideas in this field, and the first paper on backpropagation procedure on CNN was published in 1990 [22]. In 1998, LeCun wrote an overview paper on the principles of training of deep neural networks using gradient-based optimization, showing that CNN can be combined with search or inference mechanisms to model interdependent complex outputs [23]. In 2006, the Canadian Institute for Advanced Research (CIFAR) revived the interest in deep feedforward networks by connecting a group of researchers who introduced unsupervised learning procedures. The first major application of this approach was in speech recognition, which was developed during the following years; by 2012, new versions were already being deployed in Android phones. Since the 2000s, CNN have been successfully applied to the detection, recognition, and segmentation of objects/regions in images; a major recent practical success of CNN is face recognition. Despite these advances, CNN was neglected by most of the computer-vision communities until the ImageNet competition (2012) [24]. 

In recent years, we have observed the impressive emergence of complex CNNs constructed from more than 100 layers, mostly due to an increased interest in this field and initiation of a great number of scientific activities. Due to the annual software contest known as the ImageNet Large Scale Visual Recognition Challenge (ILSVRC), designated for creation of AI, a great variety of software programs have been developed, such as residual nets (e.g., AlexNet, GoogleNet, InceptionResNet, and ResNet from Microsoft and many other variants), fully convolutional networks (FCN), U-Net (which is based on an encoder–decoder mechanism for pixelwise classification and is mostly used in segmentation processing on test images [25,26,27]), and others. As foreseen by LeCun [24], human vision, natural language understanding, and major progress in AI will materialize by using systems that combine representation learning with complex reasoning.

In the area of gastroenterology, which is overwhelmed by a notably large amount of clinical data and endoscopic or ultrasound images, this technology has been applied to aid clinicians in establishing diagnosis, estimating prognosis, and analyzing images.

##### Computer Vision

Computer vision refers to the specific use of computer systems in the processing of images/videos, and the possibility of acquiring information from this processing. We must note that a multitude of technological developments have been demonstrated recently in this domain. In the medical field, clinicians work with large amounts of visual data that must be analyzed to elaborate the proper diagnosis and choice of the best treatment, especially in domains such as radiology or endoscopy. In the latter domain, CNNs were elaborated for different purposes such as esophageal/gastric cancer detection [28,29] and “real-time” polyp detection/differentiation between polyp types [30,31], among others.

### 3.3. Automated Endoscopy Report Writing 

Due to the high number of endoscopic examinations and findings, the limited storage of images during procedure, as well as the need for standardized databases for epidemiological studies, quality control, surveillance programs, and research, endoscopy reports are especially suitable for automatization and electronic storage. For that purpose, computer vision AI algorithms can be used to analyze the technical aspects of the investigation, document the activity, and enable the transfer and comparison of images and findings (coded automatically) between different hospitals or consultants. Standardized computerized report systems should be accessible, fast, and accurate, so that they can be used in the daily practice by any endoscopist. In this regard, several endoscopy software systems, such as Endobase from Olympus, have been developed in the past years, to record and store endoscopy findings and images and elaborate reports, with the goal of developing a single documentation system for the whole endoscopy workflow. These endoscopy software systems are essential for modern gastroenterology; therefore, they must be fast, informative, and comprehensive in recording and storing the endoscopy findings and to allow automatic data transfer for quality and research purposes, as well as easy data retrieval in a universally format. Furthermore, they should enable the inclusion of other crucial information, such as histopathology of detected lesions, patient’s satisfaction, adverse events, and follow-up recommendations. Moreover, they should allow database handling for many other purposes, such as safety, quality control, maintenance of equipment, management of supply, billing, and others [32,33,34].

## 4. Principal Applications of AI for Assessment of Precancerous and Cancerous Esophageal Lesions

The most important advances delivered by AI in assessment of esophageal pathology consist of screening of early esophageal carcinoma, both dysplasia/adenocarcinoma developed on Barrett’s esophagus and squamous cell carcinoma.

Esophageal carcinoma ranks seventh in terms of incidence (almost 600,000 new cases) and was also responsible for an estimated 1 in every 20 cancer deaths in 2018. Developing countries are where the histologic subtype of squamous cell carcinoma (SCC) predominates, and esophageal cancer is commonly diagnosed in an advanced stage, which is related to most of the deaths. However, adenocarcinoma (AC) represents the major histologic subtype in high-income countries, with obesity and gastroesophageal reflux disease (GERD) among the major risk factors. In recording a broad decline in the incidence of esophageal SCC, we also remark on an outburst in the incidence rates of AC, partially because of an increasing frequency of the abovementioned risk factors and perhaps also due to eradication of *Helicobacter pylori* infection. Additionally, the overall prognosis of the AC subtype remains poor, with an overall five-year survival of approximately 15% [35,36]. 

### 4.1. Identification of Dysplasia/Early Neoplasia in Barrett’s Esophagus (BE)

BE represents a major risk factor associated with the development of esophageal AC, mostly in patients with long-segment BE and in the presence of intraepithelial neoplasia [37]. Endoscopic assessment of this condition might be highly difficult, especially by nonexperts, because they must differentiate between all sequences of the carcinogenic process, namely nonneoplastic BE, low-grade/high-grade dysplasia (LGD/HGD) BE, and early adenocarcinoma (EAC). Moreover, by detecting AC in an early stage suitable to endoscopic treatment, patient prognosis might be fundamentally improved [38,39,40].

In this context, elaboration of computer assisted diagnosis (CAD) systems to detect early neoplastic areas in BE during regular endoscopic surveillance represents a priority task due to the imperative need to help endoscopists to perform accurate targeted biopsies, instead of using biopsies of any visible lesions plus random biopsies taken every 1–2 cm for the length of the BE in a four-quadrant fashion (Seattle protocol), which has proven to be a laborious, time-consuming procedure and is associated with a lower sensitivity [41,42,43]. To overcome the limitations of the procedures currently used in endoscopic surveillance of BE, the American Society for Gastrointestinal Endoscopy (ASGE) concluded that, to exclude the need for random mucosal biopsies in BE, the use of any imaging technology plus targeted biopsies should have a sensitivity of more than 90%, a specificity of 80%, and a negative predictive value of minimum 98%. However, these performance scores could be achieved only by expert endoscopists [44]. Although a large spectrum of advanced endoscopic technologies, such as magnification endoscopy (ME), chromoendoscopy (CE), probe-based confocal laser-induced endomicroscopy, endocytoscopy, volumetric laser endomicroscopy (VLE), and wide-area transepithelial sampling (WATS) with computer-assisted three-dimensional analysis, have been studied to improve BE assessment, most are expensive, time-consuming, and have a long learning curve [45,46]. Therefore, ML assistance is required for nonexpert endoscopists to perform optical diagnosis in their routine practice [44,47,48,49].

#### 4.1.1. CAD Using White-Light Endoscopy/Narrow-Band Imaging (WLE/NBI)

Van der Sommen et al. [8] constructed a CAD system for detection of early neoplasia in BE, using color filters, specific texture, and ML for WLE images, and this system was evaluated on a dataset of 100 images from 44 patients with BE. The system identified neoplastic lesions (per-image analysis) with a sensitivity/specificity of 83%. Mendel et al. [50] performed a CNN analysis of BE including 50 endoscopic WLE images of neoplastic BE and 50 noncancer images from an open access database (Endoscopic Vision Challenge MICCAI 2015), reaching a sensitivity of 94% and a specificity of 88%. From the same study group, Ebigbo et al. [51] continued research on CNN in early Barrett’s AC, using 71 high-definition WLE and NBI images of early neoplastic BE (T1a) and nondysplastic BE, achieving a sensitivity/specificity of 97%/88% (WLE) and 94%/80% (NBI) in classification of endoscopic images into cancer/noncancer types. For the MICCAI database (WLE), the results increased to a sensitivity of 92% and specificity of 100%. The model proved to be significantly more accurate than nonexpert endoscopists. In addition, Ghatwary et al. used a DL algorithm on the same open-access dataset (100 images) and achieved a sensitivity of 96% and a specificity of 92% [52].

In their pilot study, the Hashimoto group [53] constructed an AI algorithm based on a dataset including 916 WLE/virtual NBI images from 70 patients with histology-proven neoplastic BE (high-grade dysplasia/T1 cancer) in which the software masked the areas of neoplasia. Another 916 control images were collected from 30 patients with histology-proven or confocal-laser-endomicroscopy-proven normal BE. A CNN algorithm was pretrained (on ImageNet) and subsequently fine-tuned to perform binary classification of “dysplastic” or “nondysplastic”. Moreover, researchers elaborated an object detection algorithm with the goal of drawing localization boxes that surround the dysplastic regions. The CAD system evaluated 458 test images, including 225 dysplastic and 233 nondysplastic features, and was able to detect early neoplasia in BE with a high accuracy of 95.4%, a sensitivity of 96.4%, and a specificity of 94.2%. Finally, the object detection algorithm for the validation set was able to localize the areas of dysplasia with high precision (mean-average-precision of 0.7533) and at a speed that allows implementation of the model in a real-time setting.

#### 4.1.2. CAD Using Wide-Area Transepithelial Sampling (WATS)

Wide-area transepithelial sampling (WATS), associated with computer-assisted three-dimensional analysis, consists of abrasive brushing of the BE mucosa, followed by neural network analysis to identify abnormal cells. WATS is a technique that, in addition to assuring the sampling of a wide area of the BE segment, supplies a deep transepithelial section, with the aid of an abrasive brush, and appears to be associated with a high rate of interobserver agreement (overall kappa value of 0.86) [54]. The WATS^3D^ specimen is then sent to the laboratory for computer analysis that uses AI and proprietary 3D imaging to help pathologists reliably identify precancerous cells. 

Previous trials have shown that, when associated with classical biopsy, WATS leads to an increase in the detection of intestinal metaplasia and dysplasia. To this end, the multicenter prospective trial of Johanson et al. [55], performed on 1266 patients, demonstrated an increased overall detection of intestinal metaplasia by 39.8%. Anandasabapathy et al. [56], in a study on 151 patients with high-risk BE, showed that, by associating WATS to biopsy, the yield of esophageal dysplasia detection increased by 42% (additional 16 cases). We must mention that these two clinical trials were not focused on HGD/EAC and did not comply entirely with the Seattle protocol. 

A multicenter, prospective, randomized trial (www.clincaltrials.gov Clinical trial number: NCT03008980) [57] was performed on 160 referral BE patients undergoing endoscopic surveillance (using HD-WLE), with the primary aim of evaluating the use of WATS in addition to biopsy for the detection of HGD/EAC. Patients received either biopsy (according to the Seattle protocol) followed by WATS or vice versa. The rate of detection of HGD/EAC was 4.1 times higher with using WATS alone, compared with biopsy alone (29 cases vs. 7 cases), and only one case with positive biopsy was missed by WATS. The addition of WATS to biopsy led to a 14.4% increase in the detection rate of HGD/EAC, meaning 23 additional cases. Among these new cases, 11 were classified by biopsy as normal BE and 12 as LGD/indeterminate for dysplasia. The majority of these patients had prior dysplasia histories, and thus they represent a high-risk BE surveillance population. The order of procedure randomization did not influence the performance scores. The addition of WATS prolonged the procedure by an average of 4.5 min. These results demonstrate the promising role of this procedure in surveillance programs for BE.

#### 4.1.3. CAD Using Volumetric Laser Endomicroscopy (VLE)

Volumetric laser endomicroscopy (VLE) is based on use of optical coherence tomography (OCT) to generate real-time microscopic cross-sectional imaging. VLE with laser marking represents an advanced imaging technology that enables a circumferential scan of the esophageal wall layers with the aim of detecting dysplasia, and it has been commercially available in the United States since 2013. The technique supplies direct in vivo marking of suspicious areas for neoplastic transformation from which the endoscopist might collect targeted biopsies [58]. A multicenter US trial on one thousand patients reported that VLE improved the neoplasia diagnostic yield in BE by 55% [59]. Because the endoscopist must analyze an enormous quantity of complex data during this examination, computer assistance might be helpful in recognizing abnormalities. For this purpose, an AI software known as intelligent real-time image segmentation (IRIS) was developed that identifies three VLE features previously associated with histologic-proven dysplasia [60,61], namely a hyperreflective surface (marker of cellular crowding and increased nuclear-to-cytoplasmic ratio), hyporeflective structures (atypical BE epithelial glands), and the lack of a layered architecture (which differentiates between squamous epithelium and BE). This algorithm proved to be a helpful tool for detection of dysplasia during endoscopic surveillance of BE in a less burdensome manner. Swager et al. assessed the CAD detection rate of early neoplastic lesions in BE using 60 ex vivo VLE and obtained good performance cores [58], with a sensitivity of 90% and specificity of 93%. Therefore, a multicenter randomized controlled trial is currently in process to further explore the accuracy of VLE, using the IRIS program compared with VLE without IRIS (NCT03814824) [9].

Another study constructed by Struybenberg et al. [62] [NCT01862666] evaluated the performance of automatic data extraction followed by CAD analysis, using a VLE multi-frame approach for detection of BE neoplasia. Ex vivo VLE images from 29 BE-patients (nondyspastic or HGD/EAC) were retrospectively analyzed followed by assessment of sixty histopathology-correlated regions of interest (30 nondysplastic vs. 30 neoplastic) by means of different CAD systems. Furthermore, multiple neighboring VLE frames were evaluated (including 1.25 mm proximal and distal areas), and in total, the AI analysis included 3060 VLE frames. Multi-frame analysis resulted in a significantly higher median AUC, compared with single-frame analysis (used in the abovementioned study) (0.91 vs. 0.83). The multi-frame approach reached a maximum AUC of 0.94 if including 22 frames on each side of the region of interest. These data revealed rapid and accurate image interpretation and improved BE neoplasia detection by using multi-frame vs. single-frame VLE image analysis.

#### 4.1.4. CAD Using I-SCAN 

In their study, Seghal et al. [63] collected video recordings from patients with nondysplastic and dysplastic BE assessed by high-definition endoscopy, using the *i-Scan* enhancement. *I-Scan* is a revolutionary endoscopic postprocessing light filtering device that uses software algorithms equipped with real-time image mapping technology embedded with a video processor. This equipment increases the resolution above the standard high-definition level, thus offering additional features for supplementary analysis. Three image-enhancement modes are available: surface, contrast, and surface plus tone enhancement. According to the protocol, the areas of interest were recorded, and the diagnosis was histologically confirmed. The images were interpreted by three blinded experts, based on mucosal and microvasculature patterns, identification of nodularity/ulceration, and overall suspected diagnosis. These data formed the bases on the decision tree for dysplasia prediction. Subsequently, nonexpert endoscopists interpreted the same videos both before and after computer-assisted training using the previously mentioned decision tree. By assessing videos collected from 40 patients, which in 12 cases covered both before and after acetic acid application, experts obtained an average accuracy for dysplasia prediction of 88%. By entering their responses into a decision tree, the accuracy of the resultant model increased to 92%, with sensitivity and specificity of 97% and 88%, respectively. No additional improvement was obtained using acetic acid. Dysplasia detection was improved significantly in the nonexpert group after formal web-based training, reaching the accuracy obtained by experts, and sensitivity rose significantly from 71% to 83%. 

We mention a similar web-based program, previously published as an abstract presentation, designed to improve the detection of Barrett’s esophagus-related neoplasia [64]. In this study, endoscopy recordings from patients with neoplastic and nondysplastic BE were assessed by three expert endoscopists who used specialist software to delineate Barrett’s esophagus-related neoplasia (BORN) lesions. Subsequently, 68 endoscopists from the USA and Europe with different degrees of expertise were tasked with recognizing and delineating these features in four sets of 20 videos (including 48 neoplastic and 32 normal BE) with online training. A significant increase in the detection and delineation scores were observed over the four sets. After removal of 55 inadequate videos, the results of the study were validated by using a new group of 121 endoscopists across the USA, Canada, and Europe and reached similar outcomes across all levels of expertise. 

These studies highlight the usefulness of AI algorithms in accurate prediction of dysplasia and significant improvement in detection rates, as well as shortening of the learning curve once taught to nonexperts.

#### 4.1.5. Novel Research Toward Real-Time Recognition of BE

Most of the previous results related to ML-assisted evaluation of cancer in BE were achieved by using optimal endoscopic images, which might not accurately reflect the real-life context. To enable integration of CNN-based image classification into clinical practice, Ebigbo and colleagues developed a system to further increase the celerity of image analysis for classification and the resolution of dense prediction, which relies on the color-coded spatial distribution of cancer probabilities. This system represents an encoder–decoder artificial neural network that is pretrained by ImageNet and based on a ResNet containing 101 layers. The CAD system extracts random images from the real-time camera livestream during endoscopic assessment of BE performed by an expert endoscopist, thus supplying an accurate differentiation between normal BE and early esophageal AC through classification and segmentation procedures. The model was trained by using a total of 129 endoscopic images from a hospital image database. For validation, additional images (including 36 of early AC and 26 of normal BE) from 14 patients were assessed by the system during endoscopic examination. All of these images were pathologically confirmed either on resection specimens (AC) or forceps biopsies (normal BE). This CAD system, although applied to a low number of patients, offered successful real-time implementation of AI in the detection of early esophageal AC in BE in a real-life setting and demonstrated excellent performance scores, with a sensitivity of 83.7%, a specificity of 100.0%, and an overall accuracy of 89.9% [28].

The ARGOS consortium, supported by the Dutch Cancer Society and Technology Foundation STW, includes three international referral centers for detection of early neoplasia in BE, a leading academic group involved in image analysis, and two commercial enterprises that collaborate within the strategic research program known as “Technology for Oncology”. This project consisted of designing an improved version of CAD system based on high-quality endoscopic images with the goal of improving endoscopic detection of early neoplastic BE. The prospectively collected training dataset included WLE overview images of 40 neoplastic BE and 20 nondysplastic BE patients. Neoplastic images were delineated by expert endoscopists, who defined the overlap area of at least four delineations as a “sweet spot” and the area with a minimum of one delineation as a “soft spot”. The model was trained on color and texture features and assessed using leave-one-out cross-validation. Positive features were extracted from the sweet spots, and negative features were extracted from nondysplastic BE images. The system obtained an accuracy of 92% for neoplastic detection with a sensitivity of 95% and a specificity of 85%. The system delineated the soft spot and indicated the preferred biopsy location (red-flagged the area) in 100% and 90% of cases, respectively. The total time needed by the algorithm to analyze all images and delineate lesions was 61.8 s [65]. This research adds new advances toward real-time automated recognition of Barrett’s neoplasia.

### 4.2. Esophageal Squamous Cell Carcinoma

#### 4.2.1. Identification of Premalignant Lesions/Early Esophageal Squamous Cell Carcinoma (ESCC)

##### CAD Using Narrow Band Imaging (NBI)

A group of four institutions from three different countries developed a CAD system for real-time diagnosis of precancerous esophageal lesions and ESCC [10]. This model was trained by using a total of 6473 NBI images (including noncancerous lesions, precancerous lesions, and ESCC) and was validated with the aid of still endoscopic images and video images. The AI system developed a probability heat map that indicated suspected areas of neoplasia with the color yellow and noncancerous areas with the color blue. The identified neoplastic areas were masked with color. For assessment of image datasets containing 1480 malignant NBI images (59 consecutive cases), the CAD system obtained a sensitivity of 98.04%, whereas for the 5191 noncancerous NBI images (2004 cases), it obtained a specificity of 95.03%, and the area under curve was 0.989. For assessment of the video datasets of neoplastic lesions, the system obtained the following performance scores. For the 27 non-magnifying videos, the per-frame sensitivity was 60.8% and the per-lesion sensitivity was 100%. For the 20 magnifying videos, the per-frame sensitivity was 96.1% and per-lesion sensitivity was 100%. For the normal esophagus videos (including 33 videos), the model obtained a per-frame specificity of 99.9% and a per-case specificity of 90.9%. Due to the high sensitivity and specificity in recognizing precancerous lesions and ESCC in both endoscopic still images and video datasets, the DL model appears to be a helpful tool to assist endoscopists.

##### CAD Using the LASEREO System 

Recently, an image-enhanced endoscopy (IEE) system known as LASEREO (FUJIFILM Co., Japan) has emerged. This system is equipped with two laser light sources and four operating modes: white light (WLE), blue laser imaging (BLI), BLI-bright, and linked color imaging (LCI) [66,67]. Similar to NBI examination, BLI mode visualizes the microvascular and microsurface architecture of the digestive tract mucosa [68], and LCI enhances the ability to detect slight differences in mucosal color.

Non-ME endoscopic techniques are associated with a high sensitivity in identification of all lesions suspicious for esophageal SCC, whereas ME devices have a high accuracy in differentiating between cancerous and noncancerous esophageal lesions, thus offering the ability to perform a noninvasive, real-time endoscopic diagnosis known as “optical biopsy”, which reduces the need for real biopsies [69,70]. 

The Japanese group of Ohmori [71] developed a CAD system to detect and differentiate superficial esophageal SCC. The AI algorithm used a Single-Shot MultiBox Detector (SSD) containing 16 layers. After training, the CNN was validated by using the Caffe deep learning framework. Moreover, all layers of the model were fine-tuned by using weights from ImageNet. The training dataset consisted of 9591 endoscopic non-magnified/7844 ME images of 804 histology-proven superficial esophageal SCC, plus 1692 non-ME/3435 ME images from noncancerous lesions or normal esophagus. The validation dataset included 255 non-ME WLE, 268 non-ME narrow-band images/blue-laser images (NBI/BLI), and 204 ME-NBI/BLI endoscopic images collected from 135 patients. The performance of the CNN was compared with the diagnostic ability of 15 experienced endoscopists.

The AI system achieved the following performance scores for diagnosing superficial SCC. Using non-ME with WLE, the sensitivity, specificity, and accuracy were 90%, 76%, and 81%, respectively. Using non-ME with NBI/BLI, the sensitivity, specificity, and accuracy were 100%, 63%, and 77%, respectively. Using ME, the sensitivity, specificity, and accuracy were 98%, 56%, and 77%, respectively. By assessing the performance parameters together with the diagnostic flow, the CNN achieved a sensitivity, specificity, and accuracy of 98%, 68%, and 83%, respectively—results superior to those of the experienced endoscopists. Due to the high-speed analysis capacity of the system, real-time accurate diagnosis of SCC by using video images should be possible very soon.

##### Detection of Early Squamous Cell Carcinoma (ESCC) Plus ESCC Invasion Depth 

According to the Japan Esophageal Society guidelines, endoscopic resection represents a definitive indication for treating intraepithelial (EP)/lamina propria (LPM) esophageal lesions and a relative indication for muscularis mucosa (MM) lesions/cancer invading the submucosa to a depth less than 200 µm (SM1). Surgical resection/chemoradiotherapy is recommended in the case of cancer invasion of the submucosa to a depth greater than 200 µm (SM2). Therefore, accurate identification of the invasion depth is essential to avoid overtreatment and thereby improve quality of life [72]. 

The Japanese single-center retrospective study of Tokai et al. [73] assessed the capacity of an AI system to measure ESCC invasion depth. The authors used the previously developed CNN, which was initially trained on 8428 WLE/NBI images for the detection of ESCC [74]. Additionally, this preexistent system was trained using a total of 1751 new images of ESCC with information on invasion depth collected from the hospital database. Furthermore, to assess diagnostic accuracy, 291 test images were obtained from 55 consecutive patients, 42 with EP-SM1 ESCC and 13 with SM2 ESCC. These images were subsequently reviewed by both the CNN system and 13 board-certified endoscopists. The system diagnosed 95.5% (279/291) of the ESCC and predicted the invasion depth with an accuracy of 80.9% and a sensitivity of 84.1% within an interval of only several seconds. Because the CAD system presented a higher diagnostic accuracy for ESCC invasion depth vs. expert endoscopists, it could be used as an adjunctive tool in the assessment of ESCC.

##### CAD using Esophageal Intrapapillary Capillary Loops (IPCLs)

Esophageal intrapapillary capillary loops (IPCLs) are microvessels first described using magnification endoscopy (ME) [75] and represent a marker of ESCC. Changes in the morphology of IPCLs have been demonstrated to correlate with neoplastic invasion depth, a major factor in the decision of curative endoscopic therapy [76,77]. Normal IPCLs represent fine-caliber looped capillaries developed from the sub-epithelial network. During ESCC progression and destruction of the normal architecture of the esophageal wall, IPCLs become initially more tortuous and dilated and subsequently form linear dilated vascular structures associated with the appearance of avascular areas corresponding to cancer invasion deep in the mucosal layer. In the stage of deeper submucosal layer invasion, these structures obliterate and are replaced by neovascularization composed of tortuous, dilated, and nonlooped capillaries [78,79]. ME-NBI allows visualization of mucosal microvascular patterns in patients with ESCC [80]. Several classifications have been developed to categorize the abnormal changes of ICPL that correlate with histological invasion depth [76,81]. The recent Japanese Endoscopic Society (JES) IPCL classification is a simplified system [82,83] that has become popular in areas of high prevalence. Each ICPL category corresponds to a specific histological grade and invasion depth with a high accuracy of more than 90%. Additionally, JES classification is associated with excellent interobserver agreement [82,84].

Zhao et al. developed a CAD model to classify IPCL for detection/classification of SCC based on a total of 1383 lesions assessed with high-resolution endoscopes, using the ME-NBI technique [85]. The model used a double-labeling fully CNN and achieved mean diagnostic accuracies of 89.2% and 93% at the lesion and pixel levels, respectively, superior to that of endoscopists. The group of Everson [86] developed a modern AI system capable of real-time classification of IPCL morphologies as neoplastic or nonneoplastic, using ME-NBI endoscopic images. The CNN was trained by using a total of 7046 sequential high-definition ME-NBI images collected from 17 patients, including 10 ESCC and seven with normal esophagus. JES IPCL classification was performed by three expert endoscopists. The normal IPCL pattern was classified as type A, and the abnormal pattern was classified as B1–3, and for all visualized areas, histopathological assessment was obtained by two expert gastrointestinal pathologists. This CNN obtained a 93.7% accuracy (86.2% to 98.3%), 89.3% sensitivity (78.1% to 100%), and 98% specificity (92% to 99.7%) for classification of IPCL patterns as normal/abnormal. At the moment, the developed model is not yet able to categorize all of the specific subtypes with sufficient accuracy for clinical implementation. The system operates in a real-time fashion, with diagnostic prediction times between 26.17 and 37.48 ms. 

The group continued their study [87] by collecting a new dataset containing 68K binary labeled frames selected from 114 patient videos (45 normal and 69 abnormal), which were correlated to histopathology. The novel CNN algorithm fulfilled the binary classification task and explained the input features that drive the decision-making process. The method achieved an accuracy of 91.7% vs. the 94.7% reached by a group of 12 senior endoscopists, which was below the average obtained by the clinicians but still better than certain outcomes.

In the future, these novel applications could be improved and used as an in vivo clinical support tool for endoscopists in the evaluation of suspected ESCC and decision on endoscopic treatment. 

##### CAD Using the Endocytoscopic System (ECS)

The endocytoscopic system (ECS) represents a magnifying endoscopic method that allows in vivo assessment of surface epithelial cells in a real-time setting, using vital staining (e.g., methylene blue) [88,89,90]. In 2003, the first clinical trial was performed that described the characteristics of the normal surface squamous epithelium and neoplastic tissue of the esophagus [91]. Later, the characteristics of selected esophageal benign lesions were also analyzed, such as esophagitis, which is a differential diagnosis for esophageal cancer [92,93]. The ECS enables the realization of virtual histology and in vivo confirmation of histopathological diagnosis. 

Several studies evaluated the use of CAD to better discriminate neoplastic from nonneoplastic lesions of the esophagus by using ECS [94], including the model developed by Kodashima et al. [95], which enabled microscopic visualization of the mucosa, and the one developed by Shin et al. [96], which obtained a sensitivity and specificity of 87% and 97%, respectively. This model was subsequently improved by Quang et al. [97] by incorporating full automation with real-time analysis (tablet-interfaced high-resolution endomicroscopy). The new model achieved a sensitivity and specificity of 95% and 91%, respectively, and lower cost compared with laptop-interfaced systems. This software was also tested in vivo for three patients, resulting in 100% concordance with histopathological examination.

Kumagai et al. [98] proposed the use of ECS instead of biopsy-based assessment for SCC, with the aid of AI. For this purpose, the researchers designed a CNN (based on GoogLeNet) trained by using 4715 esophageal ECS images, including 1141 malignant and 3574 nonmalignant lesions. Subsequently, to evaluate the performance of the system, the group used an independent test set of 1520 ECS images from 55 consecutive patients, including 27 esophageal SCC and 28 benign lesions. The areas under the curve obtained by the CNN were 0.85 for the total images, 0.90 for higher magnification images, and 0.72 for lower magnification images. CAD obtained an overall sensitivity of 92.6% in diagnosing 25/27 SCC cases, and 25/28 benign lesions were recognized as nonmalignant, reaching a specificity of 89.3% and an accuracy of 90.9%. Two neoplastic lesions were misdiagnosed as nonmalignant by the AI but were correctly diagnosed by the endoscopist. The three cases of benign lesions diagnosed as malignant by the AI consisted of images of radiation-related esophagitis and of gastroesophageal reflux disease. Based on these promising results, CAD is expected to aid endoscopists in diagnosing SCC based on “optical biopsy” with the aid of ECS images, preferably using higher magnification pictures.

### 4.3. Esophageal Cancer Detection (SCC or AC)

The CNN developed by Horie et al. [74] used 8428 retrospectively collected WLE/NBI images of esophageal cancer histologically proven to depict SCC or AC as training dataset, including both superficial and advanced cancers, from 384 patients. To assess diagnostic accuracy, the authors used a test dataset including 1118 images from 47 patients with 49 esophageal cancers and 50 patients without esophageal cancer. The algorithm reached a sensitivity of 98% for detection of esophageal cancer. The CNN was able to detect all esophageal cancer lesions less than 10 mm in size. Although the obtained positive predictive value for test images was 40%, misdiagnosis of shadows/normal structures determined a negative predictive value of 95%. The CNN reached an accuracy of 98% in distinguishing superficial vs. advanced esophageal cancer. For the two different histologic subtypes, the accuracy in diagnosis was 99% for ESCC and 90% for EAC. These results demonstrate the ability of the constructed system to rapidly analyze a large number of stored endoscopic images with high sensitivity, leading to an improvement of early esophageal cancer detection in clinical practice in the near future (Table 1).

Numerous studies regarding the role of AI in digestive endoscopy are still preclinical and engineer-driven. Many of the presented studies are retrospective single-center that frequently show better results than what is in real settings (selection bias) and cannot analyze low-quality images. Moreover, some of them are based on traditional ML models, while the most recent ones use mainly complex DL algorithms. Moreover, in an attempt to achieve best results, researchers used different endoscopic methods, from the widely available standard endoscopy to the most advanced endoscopic techniques, which are available only in expert centers and skilled hands. In the evaluation of clinical studies, it should be mentioned that most studies were based on endoscopic still images, although the most recent efforts are made toward using more complex video sequences. We would like to point out the fact that, in the last few years, several real-life clinical studies have also been published. For the moment, many of the studies using video images are pilot studies, based on a limited number of patients. The majority of the studies presented in this paper, although showing promising results in the detection of premalignant and malignant esophageal lesions, needs further validation in prospective randomized clinical trials. Table 2 describes the existent and ongoing clinical trials using AI for diagnosing early neoplasia in Barrett’s esophagus and esophageal carcinoma (Table 2). Their results, along with the development of other real-life clinical trials, will probably help us make a step forward in defining the best CAD strategy to improve esophageal-malignancy detection. 

## 5. Future Perspectives and Challenges

Computer-assisted monitoring can assure cost-effective real-time quality control and performance of gastroscopy, and moreover, AI can offer a helpful tool for training of junior endoscopists in the future [99,100]. Another strength of such methodologies is represented by their usefulness in risk stratification of patients [2]. AI systems can aid in the detection of discrete lesions and classify suspicious lesions, thus increasing the detection rate and diagnostic accuracy of gastrointestinal lesions, especially digestive neoplasms [101]. AI algorithms reduce the workload of gastroenterologists and lead to quick and accurate diagnosis within seconds or minutes. Therefore, it is estimated that AI will assist doctors in making clinical decisions and that artificial technologies will be incorporated into routine endoscopic practice. Additionally, in clinical gastroenterology, new fields for exploration might open in the future with the support of computer-assisted diagnostics [19].

Despite the true benefits of the AI systems, several limitations still exist that must be overcome in the future [19]. First, most of the previous studies usually collect only high-quality endoscopic images to elaborate the training datasets, whereas low-quality images (in which the area of interest is covered by mucus, bile, was only partially visible, etc.) were excluded. This practice could possibly cause overfitting of the models [74,102,103] and an exaggeration of the detection accuracy. For correct assessments during endoscopy, unprocessed videos should be used in training and testing of the CNN [99,104,105]. Because most of the datasets are retrospective and usually show the most typical features of the lesion, inclusion of more atypical lesions and indicators for anatomical structures [106] might be used to improve the performance of the DL model. AI models should be assessed by using datasets and tests sets that are completely independent of the level or patient or condition and are adequately developed [16,107]. 

A highly important issue is the performance indicators for different AI algorithms, which, in previous research, have focused primarily on accuracy, sensitivity, specificity, and positive/negative predictive values, which might be influenced by the distribution of test datasets, selection bias, overfitting, or spectrum bias [99,108,109]. These confusing elements might determine overestimation of the model performance and generalization of the results; therefore, external validation using independent datasets to minimize this bias is compulsory. Many of the studies on the AI impact in gastroenterology were experimental, single center, or retrospective studies, or used specific endoscopic images available only in selected referral units. In the future, prospective multicentric randomized controlled trials with well-defined inclusion and exclusion criteria for the target population will be mandatory to demonstrate whether DL models truly mirror an improved accuracy of detection for gastrointestinal lesions in the real clinical context and to assess the impact magnitude of computer-assisted diagnosis systems in the routine workflow of endoscopists [110]. To diminish the “black box” nature of these models, i.e., their lack of explainability, and to avoid bias and achieve human acceptance, several methods, such as saliency region and attention maps, are already under development [111]. Additionally, by constructing a large number of distinct DL algorithms, all of which predict a different diagnosis, we might be confronted with a differential diagnostic dilemma. Because an increased accuracy implies a high amount of data, which is difficult to obtain due to the paucity of available medical records as a result of privacy issues, data augmentation modalities have been developed [112]. Moreover, more powerful and advanced computer algorithms, such as spiking neural networks that mimic the human brain, might become a novel scientific base for research [16,113]. The future involvement of AI in diagnostic medical procedures might also have an impact on the doctor–patient relationship, with ethical implications related to the assumption of responsibility [16]. These legal issues must be further clarified.

Bearing in mind the enormous advances in novel endoscopic devices, the increasing workload of clinicians due to the high number of patients, and implementation of endoscopic mass screening programs in certain high-risk areas for gastrointestinal malignancies, strong collaboration among physicians, researchers in the computer field, medical companies, and industry might become mandatory in the near future, for integration of powerful and advanced AI systems and algorithms into the composition of endoscopic devices and in daily clinical practice, with the aim of improving medical actions. Other problems to be solved include finding reimbursement modalities to support such advanced technologies and the development of a doctor-friendly interface for these AI systems. We should keep in mind that, in the future, it will likely be a challenging task to translate encouraging experimental research into clinical practice. However, the first steps toward publicly available large databases/platforms for further AI algorithm development and improvement have been already made [114]. As such, the future is already here.

## 6. Conclusions

Promising results show a good accuracy of CAD algorithms associated with advanced endoscopic techniques for diagnosis of esophageal carcinomas in the early and endoscopic treatable stages, which is associated with improved quality of life and better survival. Computer-assisted diagnostics quite often outperform clinician skills and might be a promising tool in the future for use of “optical biopsies” instead of difficult, time-consuming, and invasive biopsies or polypectomies. Furthermore, the novel artificial models are starting to be able to predict the depth of invasion of esophageal neoplasms with high precision and thus can help in selection and best management of tumors, such as in endoscopic vs. surgical resection. Therefore, such methodologies are useful in adoption of the best therapeutic strategy with reduced costs for healthcare systems. The link between human intelligence and artificial intelligence should evolve toward personalized medicine and, particularly, personalized gastroenterology healthcare.

## Figures and Tables

**Table 1:** Current studies applying AI in detection of esophageal cancer.

Ref.	Published Year	Aim of Study	Design of Study	Type of AI (AI Classifier)	AI Validation Methods	Number of Subjects								
						Training Dataset			Test Dataset			Performance		
						No Cases (Negative/Positive)	No Images (Negative/Positive)	Endoscopic Procedure	No Cases (Negative/Positive)	No Images (Negative/ Positive)	Endoscopic Procedure	Accuracy %	Sensitivity/Specificity%	AUC
Van der Sommen et al. [8]	2016	Detection of early neoplasia in BE	R	color filters, specific texture, and ML (“Filter with Gabor bank”, SVM)	leave- one-out CV on a per-patient basis	44 pts with BE (23/21) 100 EGD images					WLE		83 (per image); 86/87 (per patient)	-
Mendel et al. [50]	2017	Detection of early neoplasia in BE	R	CNN		50/50 EGD images (Endoscopic Vision Challenge MICCAI 2015)					HD-WLE		94/88	-
Ebigbo et al. [51]	2019	Detection of early Barrett AC	R	deep CNN (ResNet)	leave-one-patient-out CV	Local dataset: 41/33 pts, 148 HD WLE/NBI MICCAI 2015 Dataset: 22/17 pts, 100 HD-WLE					HD- WLE/NBI		Local dataset: 97/88 (WLE) 94/80 (NBI) MICCAI-dataset: 92/100 (WLE)	-
Ghatwary et al. [52]	2019	Detection of early Barrett AC	R	R-CNN, Fast R-CNN, Faster R-CNN, SSD	2- and 5-fold-CV, leave-One-Patient-Out CV	MICCAI dataset:21 pts (9/12) (training dataset)	60 (30/30) EGD images	HD-WLE	MICCAI dataset: 9 pts (4/5) (validation dataset) 9 pts (4/5) (test dataset)	40 (20/20) EGD images	HD-WLE	83 (ARR for Faster R-CNN)	96/92 (SSD)	-
Hashi-moto et al. [53]	2020	Detection of early esophageal neoplasia on BE	R	CNN based on Xception architecture, YOLO v2	Internal validation	100 pts (30/70)	1832 (916/916) EGD images	WLE/NBI	39 pts (13/26) (valida-tion dataset)	458 (233/225) EGD images (validation dataset)	WLE/NBI	95.4	96.4/ 94.2	-
Vennala-ganti et al. [57] NCT03008980	2017	Detection of early esophageal neoplasia on BE	P	neural network-based, high-speed computer scan		160 pts (134 ND/LGD, 26 HGD/EAC) randomized: −76 pts biopsy → WATS −84 pts WATS → biopsy					WATS	The addition of WATS: absolute detection rate increase 14.4%		
Swager et al. [58]	2017	Detection of early BE neoplasia	R	ML-methods: SVM, discriminant analysis, Ada-Boost, random forest, k-nearest neighbors etc.	Leave-one-out CV	−19 BE pts −60 (30/30) images					Ex vivo VLE images		90/93	0.95
Struy-benberg et al. [62] NCT01862666	2019	Detection of Barrett’s neoplasia	P	8 predictive models (e.g., SVM, random forest, Naive Bayes); best = CAD multi-frame image analysis	leave-one-out CV	−52 endoscopic resection specimens from 29 BE pts −60 (30/30) regions of interest + 25 neighboring frames → 3060 VLE frames					Ex vivo VLE images	-	-	0.94
Seghal et al. [63] UK national clinical trial (REC reference 08/H0808/8, study no. 08/0018)	2018	Detection of dysplasia arising in BE	P	ML-algorithm: DT (WEKA package)		−40 pts BE ± dysplasia					Video HD-EGD, i-Scan	92	97/88	-
Ebigbo et al. [28]	2020	Real- time detection of early neoplasia in BE	R/P	DeepLab V.3+, an encoder–decoder ANN (ResNet 101 layers)	classification (global prediction), segmentation (dense prediction)		129 EGD images	HD-WLE/ gNBI	14 pts BE (valida-tion dataset)	26/36 images (validation dataset)	random images from real-time camera livestream	89.9	83.7/ 100.0	-
De Groof et al. [65] - The ARGOS project	2019	Recognition of Barrett’s neoplasia	P	supervised ML-models (trained on color/texture features), SVM	leave-one-out CV	−60 pts (20/40) −60 EGD images					HD-WLE	92	95/85	0.92
Guo et al. [10]	2020	Real-time automated diagnosis of precancerous lesions and ESCCs	R/P	DL model: SegNet = deep encoder–decoder architecture for multi-class pixelwise segmentation	AI probability heat map-generated for each input (ESD image)	358/191 pts	6473 (3703/2770) images	NBI images	Validation: 59 consecutive cc cases (dataset A); 2004 consecutive non-cc cases (dataset B); 27 non-ME cc cases + 20 ME cc cases (dataset C); 33 normal cases (dataset D)	Validation: 1480 cc images (dataset A); 5191 non-cc images (dataset B); 27 non-ME cc images + 20 ME cc images (dataset C); 33 normal images (dataset D)	NBI images (datasets A, B); NBI video EGD images (datasets C, D)	-	98.04/ 95.03 (datasets A, B); sensitivity per- frame/lesion: 60.8/100 (non-ME video C) 96.1/100 (ME video C) specificity per frame/lesion: 99.9/ 90.9 (video D)	0.989 (data-sets A, B)
Ohmori et al. [71]	2020	Detect and differentiate esophageal SCC	R	deep Neural Network-SSD	Caffe deep learning framework	804 SSC pts	9591 non-ME/7844 ME, SCC images; 1692 non-ME/3435 ME, non-cc images	ME/non-ME ESD images	135 pts	255 non-ME WLE; 268 non-ME, NBI/BLI; 204 ME-NBI/ BLI ESD images	non-ME WLE; non-ME/ME NBI, BLI	83	98/68	-
Tokai et al. [73]	2020	Diagnostic ability of AI to measure ESCC invasion depth	R	deep neural network-SSD, GoogLeNet	Caffe deep learning framework		-pre-training 8428 images; training 1751 EGD images	WLE/NBI images	55 consecu-tive patients, 42 with EP-SM1 ESCC and 13 with SM2 ESCC	291 images	WLE/NBI images	95.5 (SCC diagnosis); 80.9 (invasion depth)	84.1 (invasion depth)	-
Zhao et al. [85]	2019	Automated classification of IPCLs to improve the detection of esophageal SCC	P	double-labelling FCN, self-transfer learning	VGG16 net architecture, 3-fold CV	−219 pts (30 inflammation, 24 LGD, 165 ESCC) −1350 images → 1383 lesions (207 type A, 970 type B1, 206 type B2)					ME-NBI images	89.2 (lesion level) 93 (pixel level)	87.0/ 84.1 (lesion level)	-
Everson et al. [86]	2019	Real-time classification of IPCL patterns in the diagnosis of ESCC	P	CNN, eCAMs (discriminative areas normal/abnormal)	five-fold CV	−17 pts (7 normal 10 ESCC) −7046 sequential HD images					ME-NBI images (Video EGD)	93.7 normal/abnormal IPCL	89.3/ 98	-
García-Peraza-Herrera et al. [87]	2020	Classify still images or video frames as normal or abnormal IPCL patterns (esophageal SCC detection)	P	CNN architecture for the binary classification task (explainability) ResNet18CAM-DS		−114 pts (45/69) −67,742 annotated frames (28,078/39,662) with an average of 593 frames per patient.					ME-NBI video	91.7	93.7/ 92.4	-
Koda-shima et al. [95]	2007	Discrimination normal/malignant esophageal tissue at the cellular level	P, ex vivo pilot	ImageJ program		−10 pts					Endocytoscopy	Difference in the mean ratio of total nuclei: 6.4 ± 1.9% in normal vs. 25.3 ± 3.8% in malignant samples		
Shin et al. [96]	2015	Diagnosis of esophageal squamous dysplasia	P	Linear discriminant analysis		−177 pts −375 sites (training set 104 sites; test set 104 sites; validation set 167 sites)					Laptop-interfaced HRME		87/ 97	-
Quang et al. [97]	2016	Diagnosis of esophageal SCC	R	Linear discriminant analysis		Data identical as for [124]					Tablet-interfaced HRME		95/ 91	-
Kumagai et al. [98]	2019	Diagnosing ESCC based on ECS images (optical biopsy)	R/P	CNN based on GoogLeNet, 22 layers-backpropagation	Cafe deep learning framework	240 pts (114/126) → 308 ECS	4715 (3574/1141) images	ECS images	55 consecutive pts (28/27)	1520 images	ECS images	90.9	92.6/ 89.3	0.85; 0.90 (HMP) 0.72 (LMP)
Horie et al. [74]	2019	Detection of esophageal cancer (SCC and AC)	R	deep CNN-SSD	Caffe deep learning framework	384 pts esophageal cc (397 lesions ESCC, 32 lesions EAC)	8428 images esophageal cc	WLE/NBI images	50/47 pts (49 lesions−41 ESCC,8 EAC)	1118 images	WLE/NBI images	98 (superficial/advanced cc) 99 for ESCC,90 for EAC	98	-
Luo et al.	2019	AI for the diagnosis of upper gastrointestinal cancers	R/P	GRAIDS: DL semantic segmentation model (encoder-decoder DeepLab’s V3 + algorithm)	internal validation, external validation (5 hospitals), prospective validation	−1,036,496 endoscopy images from 84,424 individuals used to develop and test GRAIDS					HD-WLE EGD	95.5 (internal validation set); 92.7 (prospective set); 91.5–97.7 (5 external validation sets)	94.2/92.3 (prospec-tive set)	0.966–0.990 (five external valida-tion datasets)

EGD—esophagogastroduodenoscopy; AI—artificial intelligence; R—retrospective; P—prospective; WLE—white-light endoscopy; NBI—narrow-band imaging; HD—high definition; ME—magnifying endoscopy; VLE—volumetric laser endomicroscopy; WATS—wide-area transepithelial sampling; BLI—blue laser endoscopy; ECS—endocytoscopic system; CV—cross-validation; SVM—support vector machine; ANN—artificial neural network; CNN—convolutional neural network; R-CNN—regional-based convolutional neural network; SSD—Single-Shot MultiBox Detector; FCN—fully convolutional network; DT—decision tree; ARR—average recall rate; cc—cancerous; ND—nondysplastic; LGD—low-grade dysplasia; HGD—high-grade dysplasia, EAC—early adenocarcinoma; ESCC—early squamous cell carcinoma; IPCL—intrapapillary capillary loop; eCAMs—explicit class activation maps; HRME—high-resolution microscopic endoscopy; HMP—higher-magnification picture; LMP—lower-magnification picture.

**Table 2:** Clinical trials using AI for diagnosing early neoplasia in Barrett’s esophagus and esophageal carcinoma.

Status	Study Title	Number ID/Acronym	Study Type	Conditions	Design/Interventions	Outcomes	Target Sample Size (No. Participants)	Region
Recruiting	The analysis of WATS^3D^ increased yield of Barrett’s esophagus and esophageal dysplasia	NCT03008980	Observational	GERD Barrett esophagus Esophageal dysplasia Esophagus adenocarcinoma	Diagnostic test: patients will perform routine care EGD with WATS^3D^ brush samples and forceps biopsies; collection of cytology/pathology results	**Primary** outcomes of patients undergoing WATS sampling. Specifically, incremental yield for Barrett’s esophagus and esophageal dysplasia due to WATS sampling above that noted from routine forceps biopsies in various clinical settings	75,000	US
Recruiting	Volumetric laser endomicroscopy with intelligent real-time image segmentation (IRIS)	NCT03814824	Interventional	Barrett’s esophagus with/without dysplasia Barrett’s esophagus with low/high grade dysplasia	Diagnostic test: IRIS Diagnostic test: VLE Patients will undergo a VLE exam ± IRIS per the standard of care. They will be randomized into VLE without IRIS first vs. VLE with IRIS first	**Primary:** -time for image interpretation -biopsy yield -number of biopsies	200	US
Completed	A comparison of Volumetric Laser Endomicroscopy and endoscopic mucosal resection in patients with Barrett’s dysplasia or intramucosal adenocarcinoma	NCT01862666	Observational	Barrett’s-associated dysplasia Intramucosal adenocarcinoma CAD image analysis	To evaluate the ability of physicians to use VLE to visualize HGIN/IMC in both the ex-vivo and in-vivo setting and correlate those images to standard histology of EMR specimens as the gold standard.	**Primary:** the correlation of features seen on VLE images to those seen on histopathology from EMR specimens **Secondary:** the creation of an image atlas, to determine the intra- and inter-observer agreement on VLE images in correlation with histopathology → refinement of the existing VLE image interpretation criteria and the validation of the VLE classification	30	The Netherlands
Preinitiation	The additional effect of AI support system to detect esophageal cancer-exploratory randomized control trial	UMIN 000039924/AIDEC	Interventional	Esophageal neoplasm AI	To investigate the efficacy of AI for the diagnosis of esophageal cancer	**Primary:** improvement of detection rate with AI support system in less experienced endoscopist **Secondary:** improvement of detection rate with AI support system in experienced endoscopist	300	Japan
Recruiting	Automatic diagnosis of early esophageal squamous neoplasia using pCLE with AI	NCT04136236	Observational	Esophageal neoplasm AI Confocal laser endomicroscopy	Diagnosis test: the diagnosis of AI and endoscopist	**Primary:** the diagnosis efficiency of AI for diagnosing esophageal mucosal disease on real-time pCLE examination **Secondary:** contrast the diagnosis efficiency of AI with endoscopist	60	China
Recruiting	Research on development of AI for detection and classification of upper gastrointestinal cancers in endoscopic images	UMIN000039597	Observational	Esophageal neoplasm AI	Collection of endoscopic images of upper GI cancer, development of an AI system for detection of upper GI cancer- assessment of an AI system performance by expert endoscopists	**Primary:** an accuracy of AI system for detection of upper GI cancers in endoscopic images **Secondary:** an accuracy of AI system for classification of upper GI cancers in endoscopic images	200	Japan
Completed **(April 2020)**	AI for early diagnosis of esophageal squamous cell carcinoma during optical enhancement magnifying endoscopy	NCT03759756	Observational	AI Optical enhancement endoscopy Magnifying endoscopy	Arm group label: AI visible/invisible group. The endoscopic novices analyzing the image can/cannot see the automatic diagnosis	**Primary:** the diagnosis efficiency (the sensitivity, specificity and accuracy) of the AI model	119	China

GI—gastrointestinal; AI—artificial intelligence; GERD - gastroesophageal reflux disease; EGD—esophagogastroduodenoscopy; pCLE—probe-based confocal laser endomicroscopy; VLE—volumetric laser endomicroscopy; WATS^3D^—wide-area transepithelial sampling associated with computer-assisted three-dimensional analysis; IRIS—intelligent real-time image segmentation; EMR—endoscopic mucosal resection; HGIN—high-grade intraepithelial neoplasia; IMC—intramucosal adenocarcinoma; CAD—computer-assisted diagnosis.
